# Ecological plasticity to ions concentration determines genetic response and dominance of *Anopheles coluzzii* larvae in urban coastal habitats of Central Africa

**DOI:** 10.1038/s41598-021-94258-6

**Published:** 2021-08-04

**Authors:** Neil M. Longo-Pendy, Billy Tene-Fossog, Robert E. Tawedi, Ousman Akone-Ella, Celine Toty, Nil Rahola, Jean-Jacques Braun, Nicolas Berthet, Pierre Kengne, Carlo Costantini, Diego Ayala

**Affiliations:** 1 CIRMF, Franceville, Gabon; 2 Institut de Recherches Géologiques Et Minières / Centre de Recherches Hydrologiques, Yaoundé, Cameroon; 3 MIVEGEC, Univ Montpellier, CNRS, IRD, 911 avenue Agropolis, BP 64501, 34394 Montpellier, France; 4 Géosciences Environnement Toulouse, Université de Toulouse, CNRS, IRD, Toulouse, France; 5 International Joint Laboratory DYCOFAC, IRGM-UY1-IRD, BP 1857, Yaoundé, Cameroon; 6 Institut Pasteur, Unité Environnement Et Risque Infectieux, Cellule D’Intervention Biologique D’Urgence, Paris, France

**Keywords:** Ecology, Evolution, Diseases

## Abstract

In Central Africa, the malaria vector *Anopheles coluzzii* is predominant in urban and coastal habitats. However, little is known about the environmental factors that may be involved in this process. Here, we performed an analysis of 28 physicochemical characteristics of 59 breeding sites across 5 urban and rural sites in coastal areas of Central Africa. We then modelled the relative frequency of *An. coluzzii* larvae to these physicochemical parameters in order to investigate environmental patterns. Then, we assessed the expression variation of 10 candidate genes in *An. coluzzii*, previously incriminated with insecticide resistance and osmoregulation in urban settings. Our results confirmed the ecological plasticity of *An. coluzzii* larvae to breed in a large range of aquatic conditions and its predominance in breeding sites rich in ions. Gene expression patterns were comparable between urban and rural habitats, suggesting a broad response to ions concentrations of whatever origin. Altogether, *An. coluzzii* exhibits a plastic response to occupy both coastal and urban habitats. This entails important consequences for malaria control in the context of the rapid urban expansion in Africa in the coming years.

## Introduction

In the last century, urban development has transformed the landscape worldwide^1,2^. Animals and plants living in urban settings has been able to adapt to pollution, habitat fragmentation and/or strong alteration of their habitats^3,4^. For instance, urbanization had led to the reduction or extinction of different species such as moths, ivory-billet woodpecker or rodent’s populations^5–7^. Therefore, the ability to survive and/or to proliferate under urban settings entails ecological, physiological, behavioral, and/or genetic modifications that may influence biodiversity, conservation, and health policies^8^. Distribution of disease arthropods has been also affected by urban habitats. Mosquito vectors, such as *Aedes aegypti*, *Ae. albopictus*, *Culex pipiens* and *Cx. quinquefasciatus,* are examples of how mosquitoes closely associated to human can colonize and spread across cities^9–11^. For instance, the urban expansion of *Aedes albopictus* has been associated to chikungunya virus outbreaks across the world^12,13^.

In Africa, where the urbanization rate is among the fastest in the world^14^, malaria mosquitoes have been historically confined to rural areas because *Anopheles* larval development is strongly hindered in polluted water^11,15^. For this reason, previous entomological and epidemiological studies predicted that urban expansion in Africa would reduce malaria transmission^11,15–17^. Indeed, despite their favourable conditions in terms of blood meal opportunities and aquatic resources, African cities have been oases of reduced malaria transmission due to in part by the limited suitable vector breeding sites^18,19^. However, in the last decades, many works have recorded *Anopheles* presence in towns across the continent^20–27^. This caused alarm in public health community because *Anopheles* proliferation might lead to a risk of malaria transmission in a population with low or absent immunity to human malaria parasites^18,28–31^. The most abundant malaria mosquitoes in urban settings are members of the *Anopheles gambiae* complex^32^. Among them, *Anopheles coluzzii* is the predominant mosquito in cities of Central Africa^20,22,33–35^, whereas *Anopheles arabiensis* has been recorded in the cities of the West Africa^36–38^. Moreover, urban malaria expansion could also be promoted by the recent and troubling urban invasion by *Anopheles stephensi* in Sudan and Ethiopia^39–41^. In Central Africa, empirical studies revealed that *An. coluzzii* is more tolerant to ammonia concentrations than *An. gambiae*^42,43^. Ammonia is very abundant in urban settings, related to organic compounds ^44,45^. Therefore, the presence of this mosquito could be linked to its higher ability to breed in most polluted breeding sites of cities^24,42,46–49^.

Recently, Cassone et al., investigated the genetic basis of the presence of *An. coluzzii* in urban settings by transcriptomic analysis of larvae breeding in polluted sites of the city of Yaoundé (Cameroon)^50^. They found that genes related to oxidative stress and detoxification were upregulated in those larvae. Moreover, Antonio-Nkondjio and co-workers reported the association between *An. coluzzii* larvae presence in polluted sites and resistance to insecticides of adults in the cities of Yaoundé and Douala^46^, leading to the hypothesis that anthropogenic pressures have conditioned the evolution of this main malaria vector^51^. Interestingly, *An. coluzzii* is also more abundant than *An. gambiae* in coastal habitats due to its higher tolerance to salinity concentrations^34,52^. However, it is not known whether ammonia and salinity tolerance share a common physiological mechanism origin in this malaria mosquito. Although, the ability of this mosquito to colonize breeding sites with higher concentrations of salts and ammonia lead to hypothesize that similar ecological and genetic factors are incriminated.

To better understand the ecological and genetic drivers involved in the colonization of breeding sites by *An. coluzzii*, we performed a physicochemical analysis of the breeding across five coastal sites (rural and urban) in Cameroon and Gabon. Our study revealed the complexity and diversity of the breeding sites colonized by *An. coluzzii*, highlighting the role of some biotic and abiotic factors in the species distribution. Nonetheless, transcriptomic analysis of ten candidate’s genes involved in detoxification and osmoregulation revealed comparable levels across breeding sites analyzed, suggesting a common genetic response against different ions presence. These results show the ability of *An. coluzzii* to colonize a broad number of aquatic conditions, with direct consequences for malaria control in urban settings.

## Results

### Breeding site diversity

**Figure 1:**
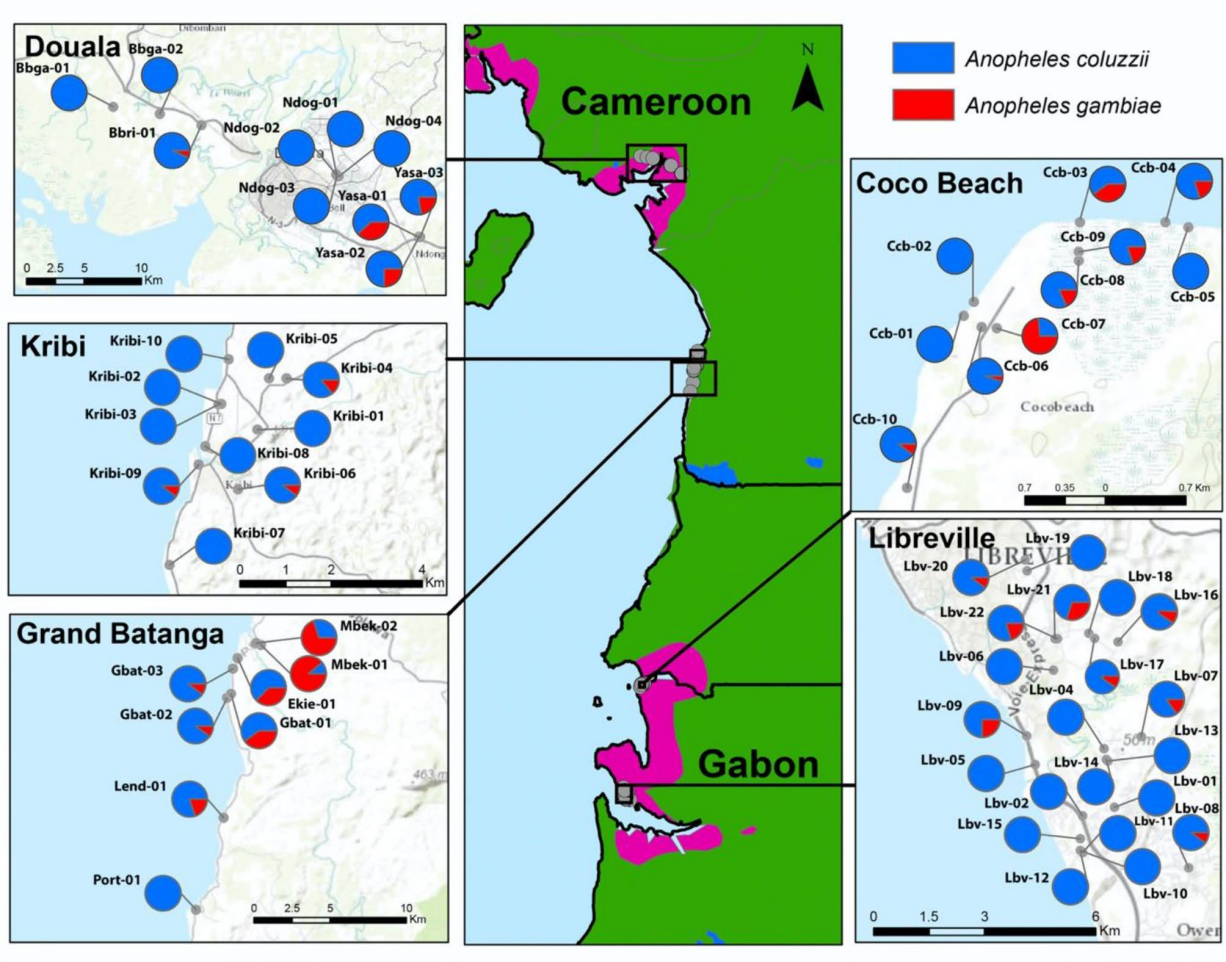
Regional distribution of *An. coluzzii* and *An. gambiae* in the collection sites.

**Figure 2:**
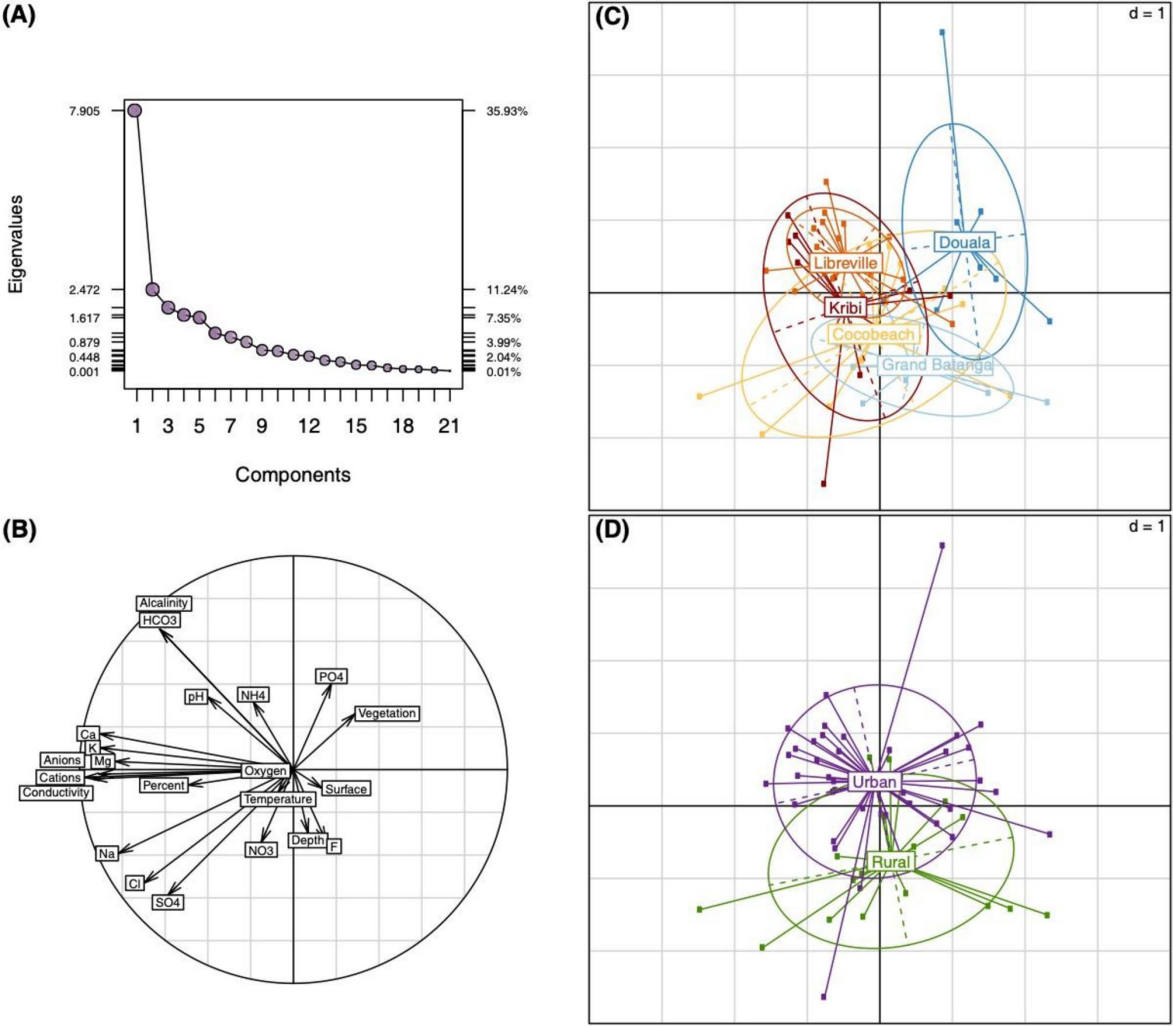
Breeding sites characterization according to the physico-chemical variables. (**A**) Principal components selected with the Broken Stick model. (**B**) Canonical weight of each variable on the principal components; the arrow size indicates the importance of the parameter. (**C**) and (**D**) PCA diagrams showing the correlations between rural and urban breeding sites and physicochemical characteristics.

In this study, 59 breeding sites and 1351 larvae were analyzed at five localities in Cameroon and Gabon (Fig. 1, Tables 1, and S1) that display great diversity in terms of the values of physicochemical variables and types of settings (Table 2, Fig. S1, Table S1). The Principal Components Analysis (PCA) selected the first two principal components (PCs) using the broken stick model test ^53^ (Fig. 2A). These two PCs accounted for 47.17% of the total variance. PC1 (35.93%) can be interpret as a salinity gradient of breeding sites, characterized by sodium (Na^+^) and chlorine (Cl^−^) concentrations (Fig. 2B). PC2 (11.24%) was correlated with physicochemical variables associated to organic pollution, characterized by concentrations of ammonia (NH_4_^+^) and phosphates (PO_4_^−^) and pH (Fig. 2B). When the different breeding sites were plotted per locality, the PCA showed a partial overlapping, with Douala as the most differentiated locality (Fig. 2C). On the other hand, the PCA analysis allowed discriminating between urban and rural settings (Fig. 2D). The segregation between these settings was higher along PC2 (organic pollutant gradient) than PC1 (salinity gradient) (Fig. 2D). The analysis of the 22 parameters measured in this study revealed that the concentration of alkalinity, calcium (Ca^2+^), NH_4_^+^, carbonate (HCO_3_^−^), significantly varied between rural and urban habitats (Table 2). Analysis of these parameters among sites showed significant variations in concentrations, for instance, of F^−^, Ca^2+^, alkalinity, Cl^-^ and HCO_3_^−^ (Table 2). Other variables, such as NH_4_^+^ and nitrates (NO_3_^−^), were comparable among sites^54–56^. Finally, analysis of the differences among sites colonized by *Culex* species, a biomarker of organic pollution^11,57–59^, revealed that only the presence vegetation significantly varied between sites colonized or not by *Culex* species (Table 2).

**Table 1:** Sampling locations and number of samples used in this study.

Country	Locality	Habitat	Sites	Larvae	*An. coluzzii*	*An. gambiae*	Hybrid
Cameroon	Douala	Urban	10	254	92.12 [60.0–100]	7.10 [0–38.2]	0.78 [0–20]
Cameroon	Kribi	Urban	10	150	95.33 [85.7–100]	4.67 [0–14.3]	0
Cameroon	Grand Batanga	Rural	8	165	69.70 [11.1–100]	29.7 [0–37.5]	0.6 [0–2.4]
Gabon	Libreville	Urban	21	474	95.78 [75.0–100]	4.22 [0–15.4]	0
Gabon	Cocobeach	Rural	10	308	76.30 [25.6–100]	23.40 [0–71.8]	0.3 [0–2.6]

Sites, number of breeding sites at each locality. Larvae, total number of samples analyzed (modelling and genomic analyses). *An. coluzzii,* mean frequency of this species per locality; in brackets the minimum and maximum frequencies.

**Table 2:** Effect of the habitat on the physicochemical parameters of breeding sites.

Variable	Total	Habitat			Sites						*Culex*		
		Rural	Urban	*P value*	Libreville	Cocobeach	Douala	Kribi	Grand Batanga	*P value*	Absence	Presence	*P value*
Alkalinity (μEq/L)	1470 ± 152.19	797 ± 59.63	1765 ± 164.69	**0.003**	2098 ± 108.58	765 ± 58.72	829 ± 96.60	2002 ± 251.13	836 ± 64.57	** < 0.001**	1411 ± 148.42	1544 ± 159.35	0.79
Anions (mg/L)	2740 ± 394.73	3315 ± 648.08	2488 ± 209.34	0.500	2767 ± 131.88	4484 ± 844.54	957 ± 91.78	3432 ± 289.15	1855 ± 168.72	0.087	2497 ± 229.91	3049 ± 539.24	0.87
Ca^2+^ (mg/L)	33.50 ± 3.31	23.70 ± 3.11	37.70 ± 3.27	**0.023**	44.20 ± 2.54	28.60 ± 3.84	12.70 ± 0.86	49.10 ± 4.06	17.60 ± 1.82	** < 0.001**	33.80 ± 3.63	33 ± 2.90	0.67
Cations (mg/L)	4329 ± 1080.44	6576 ± 1903.88	3342 ± 310.76	0.290	4036 ± 275.48	10,271 ± 2498.33	962 ± 85.40	4267 ± 333.93	1957 ± 185.26	0.11	4823 ± 1360.47	3702 ± 571.52	0.89
Cl^−^ (mg/L)	29.10 ± 12.37	71 ± 21.61	10.70 ± 1.76	0.210	12.30 ± 1.85	120 ± 28	1.22 ± 0.19	16.80 ± 1.86	10.20 ± 1.46	**0.02**	23 ± 6.18	36.80 ± 17.45	1
Conductivity (µS/cm)	549 ± 162.60	954 ± 289.41	371 ± 30.33	0.36	446 ± 27.34	1528 ± 379.24	142 ± 18.36	442 ± 28.38	236 ± 21.48	0.088	625 ± 204.40	451 ± 86.84	0.82
Depth (cm)	13.20 ± 1.26	13.50 ± 1.06	13.10 ± 1.35	0.59	16.50 ± 1.76	11.90 ± 0.53	9 ± 0.32	10 ± 0.48	15.50 ± 1.50	0.2	10.50 ± 0.51	16.60 ± 1.73	0.15
F^−^ (mg/L)	0.16 ± 0.03	0.33 ± 0.04	0.09 ± 0.01	0.077	0.09 ± 0.01	0.11 ± 0.02	0.05 ± 0.00	0.11 ± 0.02	0.60 ± 0.04	** < 0.001**	0.17 ± 0.03	0.15 ± 0.03	0.81
HCO_3_^−^ (mg/L)	89.70 ± 9.28	48.60 ± 3.65	108 ± 10.05	**0.003**	128 ± 6.63	46.70 ± 3.58	50.60 ± 5.88	122 ± 15.36	51 ± 3.94	**0.007**	86.10 ± 9.05	94.20 ± 9.73	0.67
K^+^ (mg/L)	11.70 ± 1.74	9.41 ± 1.89	12.80 ± 1.68	0.21	16.30 ± 1.81	13.40 ± 2.45	4.59 ± 1.21	13.50 ± 1.45	4.47 ± 0.38	0.087	11.50 ± 1.94	12 ± 1.50	0.42
Mg^2+^ (mg/L)	13.70 ± 5.90	29.40 ± 10.51	6.78 ± 0.90	0.14	9.56 ± 1.04	52.10 ± 13.67	1.36 ± 0.17	6.36 ± 0.51	1.14 ± 0.10	0.055	16 ± 7.43	10.70 ± 3.09	0.28
Na^+^ (mg/L)	25.30 ± 11.04	61.60 ± 19.40	9.30 ± 1.33	0.15	9.13 ± 1.06	95.20 ± 25.77	1.70 ± 0.23	17.20 ± 1.74	19.60 ± 2.14	0.07	31.30 ± 13.93	17.50 ± 5.60	0.59
NH_4_^+^ (mg/L)	2.13 ± 0.59	0.33 ± 0.07	2.93 ± 0.69	**0.007**	3.80 ± 0.83	0.31 ± 0.09	0.46 ± 0.08	3.55 ± 0.63	0.36 ± 0.04	0.088	2.53 ± 0.69	1.63 ± 0.45	0.56
NO_3_^−^ (mg/L)	0.77 ± 0.35	1.32 ± 0.48	0.52 ± 0.27	0.67	0.13 ± 0.05	1.58 ± 0.65	0.32 ± 0.09	1.54 ± 0.54	1 ± 0.12	0.52	0.73 ± 0.36	0.81 ± 0.34	0.36
Oxygen (%)	79 ± 3.25	83 ± 3.71	77.30 ± 3.06	0.89	74.20 ± 3.83	94.10 ± 4.30	83.70 ± 0.68	77.10 ± 2.73	69.3 ± 1.74	0.2	81.20 ± 2.76	76.30 ± 3.83	0.47
Percent (%)	10.30 ± 2.53	8.40 ± 2.70	11.10 ± 2.47	0.26	16.40 ± 1.21	16.10 ± 3.01	1.14 ± 3.81	9.93 ± 2.54	-1.18 ± 1.69	0.087	10.80 ± 2.71	9.60 ± 2.33	0.81
pH	7.65 ± 0.10	7.44 ± 0.11	7.74 ± 0.09	0.32	7.79 ± 0.06	7.30 ± 0.10	7.71 ± 0.11	7.66 ± 0.12	7.62 ± 0.12	0.56	7.78 ± 0.09	7.48 ± 0.10	0.19
PO_4_^3−^ (mg/L)	0.33 ± 0.30	0	0.48 ± 0.36	0.13	0.05 ± 0.02	0	1.86 ± 0.73	0	0	0.27	0.02 ± 0.01	0.73 ± 0.46	0.43
SO_4_^2−^ (mg/L)	19.70 ± 5.04	21.80 ± 4.30	18.80 ± 5.38	0.62	14.90 ± 1.56	13 ± 3	1.32 ± 0.13	44.40 ± 10.22	32.90 ± 5.39	0.09	19.70 ± 3.53	19.80 ± 6.56	0.5
Surface (m2)	1.46 ± 0.30	2.16 ± 0.46	1.15 ± 0.18	0.580	1.52 ± 0.22	1.67 ± 0.33	0.55 ± 0.05	0.98 ± 0.17	2.77 ± 0.60	0.32	1.34 ± 0.25	1.61 ± 0.36	0.63
Temperature (°C)	29.80 ± 0.34	29.60 ± 0.38	29.80 ± 0.32	0.61	29 ± 0.26	28.60 ± 0.29	29.50 ± 0.30	31.90 ± 0.33	30.90 ± 0.42	**0.01**	30.0 ± 0.38	29.40 ± 0.27	0.53
Vegetation (%)	0.15 ± 0.03	0.12 ± 0.02	0.16 ± 0.04	0.79	0.24 ± 0.04	0.10 ± 0.02	0.04 ± 0.01	0.11 ± 0.03	0.14 ± 0.02	0.22	0.09 ± 0.02	0.22 ± 0.04	**0.017**

Data are the mean ± standard error; *P-values* were estimated with the Fisher’s exact test. Significant *P-values* are in bold. See Table S1 for the complete list of variables at each breeding site.

### Spatial distribution and ecological modelling of An. coluzzii in coastal sites

**Figure 3:**
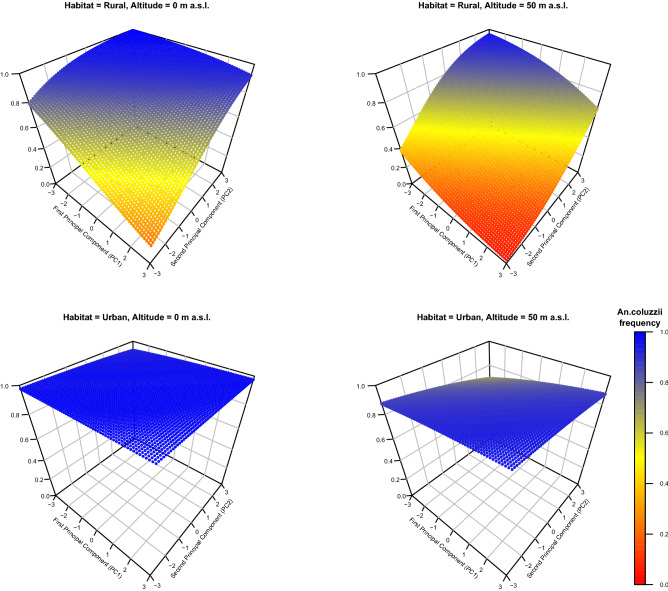
Association between environment type and altitude and *An. coluzzii* frequency.

Molecular species identification of 1351 *Anopheles* larvae from the 59 breeding sites (Table 1, Table S1) showed that *An. coluzzii* was the predominant species (n = 1181), compared with *An. gambiae* (n = 166) (Fig. 1). Only four hybrids were found and removed for further analysis (Table S1). *Anopheles coluzzii* frequency varied among localities, decreasing from urban (Libreville: 95.78%; Kribi: 95.33%; Douala: 92.13%) to rural areas (Cocobeach: 76.30%; Grand Batanga: 69.70%) (Table 1, Fig. 1). The differences in *An. coluzzii* frequency were not significant among locality (ANOVA, F = 1.179, *P-value* > 0.05), and among habitats (ANOVA, F = 0.045, *P-value* > 0.05). Our ecological modelling approach retained the first two PCs, habitat (urban or rural), altitude, and presence of *Culex spp* as significant predictors to explain the relative *An. coluzzii* frequency variation across localities and breeding sites (Table 3). Moreover, the best model (with ΔAIC = 0, Akaike Weight = 0.36) showed that the interactions between PC1/PC2 and habitat and *Culex* were significant (Table 3). Our results revealed that in urban settings, *An. coluzzii* frequency increased with high concentrations of mineral salts and organic matter, thus confirming its higher tolerance to salts and ammonia^42,52^. Moreover, altitude, which was correlated to distance to the sea level and salinity gradient (PC1) (r = 0.39, *P-*value < 0.05), greatly influenced *An. coluzzii* frequency compared with *An. gambiae* in rural habitats, but not in urban settings (Fig. 3).

**Table 3:** Result of the generalized mixed linear models to explain the absolute frequency of *An. coluzzii* at the different breeding sites.

Model No. (rank)	Fixed effects										Df	ΔAIC	Akaike weight
	Intercept	PC1	PC2	Habitat	Altitude	*Culex*	Habitat × PC1	Habitat × PC2	*Culex* × PC1	*Culex* × PC2			
**480 (1)**	2.233	-0.388	0.912	+	-0.044	+	+	+		+	11	0	0.36
**414 (2)**	1.924	-0.418	0.615	+	-0.036		+	+			9	0.66	0.259
**512 (3)**	2.201	-0.479	0.881	+	-0.045	+	+	+	+	+	12	0.74	0.249

PC1 = Principal Component 1, extracted from the results of the Principal Component Analysis (PCA); PC2 = Principal Component 2, extracted from the PCA results; Habitat = Urban gradient, with two levels: urban (Libreville, Douala and Kribi) and rural (Cocobeach and Grand Batanga); *Culex* = presence or absence of *Culex spp*. larvae; +  = term significantly retained in the model; Intercept (logit scale) = fitted value for baseline factor levels. In all models, random effects are breeding sites within each locality.

### Genetic response to habitats and environmental stressors

**Figure 4:**
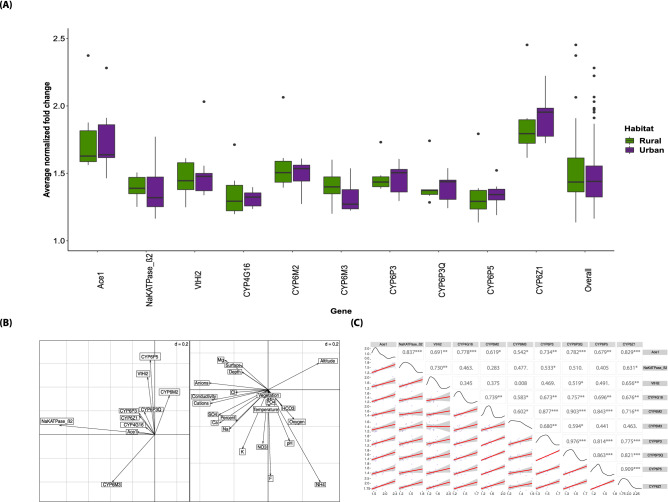
Correlation between gene expression profiles, habitat (rural and urban) and environmental predictors. (**A**) Association between habitat type and expression for each gene. (**B**) Coinertia diagram between gene expression on the left, and environmental predictors on the right. (**C**) Correlogram between genes (“number” = correlation coefficients; significance code 0’***’, 0.001'**', 0.01'*', 0.05'.' and 0.1' '; each blue cross (*) corresponds to a site).

Finally, we assessed the expression of eight genes implicated in insecticide resistance (six genes of the CYP6 subfamily^62,63^, CYP4G16^64,65^, and acetylcholinesterase 1(Ace-1)^62^) and of two genes involved in osmoregulation (Na/K transporting ATPase and V-type H + -transporting ATPase subunit 1^66–69^) (Table S2) in mosquitoes collected at 15 breeding sites across the urban (Douala; n = 3; Libreville n = 6) and rural settings (Grand Batanga; n = 2; Cocobeach n = 4). After the “Bonferroni” correction, the comparison of their expression profile did not show any significant difference between habitats (Wilcoxon test, *P-value* > 0.05) (Fig. 4A), despite the large gene expression variations across sites (Figs. S2, S3). Although, all genes under study tended to be strongly expressed, only CYP6Z1 and Ace1 showed significantly higher fold changes than the other genes (t test, *P-value* < 0.05, after Bonferroni correction) (Figs. S2, S3). As most of these genes, particularly those belonging to the CYP6 subfamily, have been repeatedly involved in insecticide resistance in *An. gambiae* in different parts of Africa^62,63,70–74^, and in other *Anopheles* species, such as *Anopheles funestus*^75^, *Anopheles minimus*^76^ and *Anopheles sinensis*^76^. The insecticide resistance status (i.e. presence of the kdr-West mutation) was investigated in a subsample of 80 mosquitoes from the five localities. Overall, 89% of these mosquitoes were homozygotes for the resistant alleles (Table S3). This indicates that the kdr mutation is presumably almost fixed across localities, thus possibly explaining the absence of expression differences between habitats (Fig. 4A), and among sites (Figs. S2 and S3)^63^. In order to investigate the environmental patterns of genes expression, we performed a coinertia analysis between the set of environmental predictors and the genes expressions changes of the studied genes. Our results identified co-relationships between most of the genes involved in insecticide resistance and the ions of anthropogenic origin (Fig. 4B). Only, the NaKATPase_β2 gene involved in osmoregulation processes, was highly correlated with the salinity (Fig. 4B). These results were confirmed by a correlation analysis among genes. All the expression patterns of gene involved in insecticide resistance were highly correlated (Fig. 4C), with the exception of the NaKATPase_β2 gene.

## Discussion

During the last decades, an increasing number of studies reported the presence of *Anopheles* in the African cities^20–27^. The present study investigated a large number of the ecological characteristics of the breeding sites exploited by *An. coluzzii* in rural and urban sites across coastal habitats of Central Africa, where this species is predominant compared with its sister species *An. gambiae*^34,42,52^. Three results emerge from our study. First, *An. coluzzii* shows an ability to colonize a large variety of water habitats, from natural ones to more anthropogenic areas. Second, *An. coluzzii* is more prone to breed in sites with higher concentrations of ions than *An. gambiae,* whatever the urbanization degree^77,78^. Third, gene expression patterns of the ten candidate’s genes in *An. coluzzii* are comparable between urban and rural habitats, suggesting that *An. coluzzii* exhibits a general response to the ions presents in each breeding site rather than specific responses to urban pollutants and to environmental stress. Altogether, our study suggests that *An. coluzzii* adaptation to urban settings is rooted in its ability to exploit more ions rich aquatic habitats.

*Anopheles coluzzii* ecological success is illustrated by its phenotypic plasticity^34,77,78^. At the larval stage, this mosquito exhibits an extraordinary adaptability to develop in a great variety of breeding sites^22,79–82^, with a preference for more permanent ones^83^. Our study confirmed the wide range of aquatic conditions where this species can lay and develop, in coastal and urban settings of Central Africa. Concerning the physicochemical characteristics of the breeding sites, our results were similar to those of previous studies in Cameroon^84^ and Kenya^85^, except for conductivity, because they concern inland sites (Table 2). Compared with published findings from other urban coastal sites in West Africa, we found lower concentrations of pollutants, such as NH_4_^+^ and NO_3_^–86^, revealing that *An. coluzzii* can still colonize breeding sites with a higher concentration of these ions (Table 2). We recorded the presence of *Culex*. The most abundant *Culex* species in the cities, *Cx. quinquefasciatus*, exhibits a high tolerance for organic matter but very low tolerance to salinity^11,52,57–59^, therefore, being a good biomarker of high ammonia and low salts concentrations. In this sense, Kudom et al*.,* 2015 observed higher NH_4_^+^ concentration and lower conductivity at breeding sites colonized by *Culex quinquefasciatus*^80^. We did not observe any significant difference of NH_4_^+^ concentration between breeding sites with/without *Culex*. Moreover, we found a large variation of physicochemical parameters among and within sites. Cities are not uniform habitats because they combine multiple and heterogeneous ecological settings^3,87^. From their origins, African towns have displayed different degrees of urbanization^88^. This feature may help mosquitoes that are less tolerant to water pollutants to survive and breed within cities. For instance, we recorded the presence of *An. gambiae* in our three urban settings (Libreville, Douala and Kribi). *Anopheles gambiae* has already been recorded in other cities of Central and West Africa. Its relative frequency varies from absence in Yaoundé and Douala (Cameroon)^22,84^ to predominant in Bangui (Central Africa Republic)^89^, and intermediate in Ghana^90^. Therefore, it would be interesting to perform phenotypic experiments across cities, including larval transplantation, in order to investigate survival, physiological changes or behavioral response to predation, for instance, in both species^91,92^. Unfortunately, the temporal physico-chemical characteristics were not considered in the present study. Important variations could occur across the year, which may provide key information at a very fine scale to better understand frequency changes between *An. gambiae* and *An. coluzzii* and therefore, urban local adaptation drivers^93^.

We then analyzed the distribution of *An. coluzzii* relative to its sister species *An. gambiae*. We observed a positive and significant association of *An. coluzzii* relative frequency with ion concentrations, represented by PC1 and PC2 (Fig. 2B, Table 3). PC1 may be interpreted as a salinity gradient (i.e. conductivity), while PC2 can be interpreted as a human pollution gradient because correlation with concentrations of NH_4_^+^ and PO_4_^−^. Our results are in agreement with previous phenotypic experiments that revealed *An. coluzzii* higher tolerance to ammonia and salinity^42,43,52^, in congruence with, previous studies in Burkina Faso that showed higher *An. coluzzii* frequency in more anthropogenic habitats^83^. However, it is not clear if tolerance to different ions has the same physiological origin. In aquatic arthropods, including *Anopheles* species, Na^+^ and K^+^ ions are actively transported via the Na/K pump^94^. This physiologic mechanism could also be involved in NH_4_^+^ transport through interaction with the potassium binding site of the pump, as observed in other organisms^95–97^. For instance, future studies will study how the inhibition of the Na/K pump could affect the tolerance to salinity and ammonium in *Anopheles*. The habitat type (urban vs. rural) also had a significant effect on the relative frequency of *An. coluzzii*, showing that there is an ecological segregation between *An. coluzzii* and *An. gambiae*. In coastal and urban habitats of Central Africa, *An. coluzzii* remains the predominant species, while *An. gambiae* dominates the rural areas^22,34^. This distribution pattern has been also observed through the cities of West-Central Africa, such as Accra^90^, Cotonou^98^, Abidjan^99^ or Luanda^100^, while in inland cities, other species are more prone to proliferate, such as *An. arabiensis* in Bobo-Dioulasso^101^. It leads to think that distance to coast is associated to the predominance of *Anopheles coluzzii*, with the exception of Dakar, where *An. arabiensis* is the predominant species^102^. In addition, our modelling approach revealed the influence of lower altitude on *An. coluzzii* frequency in rural, but not in urban habitats. In coastal areas, altitude is related to the distance to the sea, and therefore it is negatively correlated with conductivity (r = − 0.16), Na^+^ (r = -0.15), and Cl^−^ (r = − 0.15). The observation of this pattern in urban areas suggests that other factors, such as organic pollution, play a more important role. Finally, the model reveals the co-occurrence of *Culex* species and *An. coluzzii* (Table 3). The fact that *An. coluzzii* is significantly found in the same breeding sites as *Culex*, probably *Cx. quinquefasciatus*^20,103–105^ that is known to breed in larval habitats rich in decomposing organic matter^22,106,107^, suggests that this mosquito may be developing mechanisms to tolerate organic stress. However, the quantification of other parameters such as oxygen demand (COD) or biochemical oxygen demand (BOD) should be consider in order to improve the characterization of organic pollution in the breeding sites^108–110^. In addition, nitrites (or nitrates)^46,48^ and heavy metals that reveal pollution related to human activities such as agriculture, energy conversion and industry could potential provide clues of the urban pollution nature^111^.

To better understand the proliferation process of *Anopheles* in urban settings, we then compared the gene expression of ten candidate genes involved in detoxification and osmoregulation^62,63,70,112^ (Table S2) from specimens collected in urban and rural coastal sites. Our results did not find any significant difference between habitats, although all genes tended to be overexpressed with regard to the house-keeping gene used in this study. Associated with this result, the expression of each gene is highly correlated with that of the other nine genes. These results are consistent with a common response to high ion concentrations, of whatever origin. The function of these genes could be influenced by the physicochemical parameters of each breeding site. In the town of Accra (Ghana), King et al*.,* studied the correlation of the expression of five genes with the different physicochemical variables of the breeding sites^86^, and found a significant positive correlation of all genes with Ca^2+^ levels. In the same sense, our co-inertia analysis showed a strong correlation between most of the genes involved in insecticide resistance and ions (Ca^2+^ and F^−^). In larvae of Bombyx mori (the silkworm), exposure to F- pollution is strongly correlated with overexpression of cytochrome oxidases belonging to the CYP6 (CYP6AB4, CYP6AE and CYP6B29) and CYP4 (CYP4M5, CYP4M9) subfamilies^113^. Thus, gene expression could be the result of a specific ionic signal or the interaction between several genes. Also, this observation confirms the fact that there are interactions between tolerance to environmental chemicals present in the breeding sites and insecticide resistance in *Anopheles*^51,114,115^. Indeed, several studies mention the involvement of the cytochrome oxidase P450 family in the detoxification of insecticide molecules and pollutants present in the environment^63,86,116–118^. Also, the NaKATPase_β2 gene, related to the Na/K pump, was found to be particularly strongly correlated with salinity. This pump has been already incriminated in the response to salinity in mosquitoes of the genus *Aedes*^119^ and the genus *Anopheles*, particularly *An. merus*^112^. Finally, the correlogram between the different genes showed a significant correlation between genes involved in insecticide resistance and those involved in osmoregulation, mainly VtHi2. This result may suggest a possible overlap or interaction between osmoregulation and detoxification in the immature forms of *An. coluzzii*. Overall, these results reveal a global pattern of enzyme activities in response to the ions studied at the breeding sites^86^, particularly of genes involved in detoxification, such as the P450 family.

## Conclusion

During its history, *An. coluzz*ii has strengthened its deadly relationship with humans by taking advantage of anthropogenic activities, such as the agriculture^83,120^, up to the colonization of urban environments^22,48,121^. In Central Africa, our study confirmed the ecological plasticity of this species that can colonize a large spectrum of ion concentrations. Compared with its sister species *An. gambiae, An. coluzzii* has become predominant in more ion rich habitats, such as coastal and urbans areas. These results raise important questions about the origin and evolution of its osmoregulatory capacities^52^. Africa is undergoing a large transformation through a fast urbanization^14^. The colonization of this habitat by *Anopheles* could have important health consequences. For this reason, it is important to determine the fitness cost of this adaptation, how the vector competence will evolve, and what genes are involved in the underlying ecological and behavioral mechanisms. Finally, our study highlighted a similar gene expression profile of specimens coming from rural and urban habitats, which are characterized by exposure to different ions. Future studies should focus on predicting the risk of malaria transmission according to the specific socio-economic and ecological characteristics of the urban settings. This is a priority to achieve the goal of malaria control in Africa in the coming decades.

## Material and methods

### Study sites and larval collections

Mosquito larvae were collected in September 2014 and January 2015 in Cameroon and Gabon, respectively. Coastal sites were selected in urban and rural habitats (Table 1). In Cameroon, sampling was carried in Douala, Kribi and Grand Batanga. Douala (urban), the economic capital of Cameroon, presents the highest urbanization level in the country. The locality has a flat topography, making difficult the discharge of wastewater. Kribi (urban) is a port town in the southern region of the country, with a rapid demographic and urban growth due to economic development. Grand Batanga (rural) is a coastal village, south of Kribi where the main activities are fishing and plantations. In Gabon, sampling was carried out in Libreville and Cocobeach. Libreville (urban) is located along the northwest coast of the country. It is characterized by rapid population growth, associated with haphazard urban development and poor management of waste produced by human activities, particularly at its periphery. Cocobeach (rural) is a locality with moderate population growth and low human activities (mainly fishing).

Potential breeding sites were systematically inspected to determine the presence of *Anopheles* larvae^122^. To standardize the genetic analyses, larvae were collected from 7.30 am to 11.30 am using the dipping technique^50,122^. At each breeding site, four pools of ten *Anopheles* larvae at stage L3-L4 were collected. The first pool was stored in 1 ml of 100% ethanol for molecular analysis to species identification. The other pools were stored in 2 ml of RNAlater (Qiagen), for subsequent expression analysis. All samples were stored at -20 °C.

### Physicochemical characteristics of the breeding sites

The physical and chemical characteristics of the larval habitats were investigated using in situ and ex situ measurements. In situ measures characterized the parameters related to the physicochemical characteristics of the breeding sites, such as hydrogen potential (pH), conductivity (μS/cm), dissolved oxygen (%), temperature (°C) and salinity (mg/l), using a WTW 3110 instruments. Ordinal variables, such as turbidity (nothing, low, way and high), percentage of vegetation, mosquito larval density (low, way and high), and categorical variables, such as the presence/absence of mosquito larvae of the *Culex* genus*,* and breeding site physiognomy (puddle, drainage channel, canoe, etc.), were also investigated (Table S1 and Fig. S1). Ex situ measures were carried out at the Geochemical Water Analysis Laboratory (LAGE/CRH-IRGM) of Yaoundé, Cameroon. To this aim, 200 mL of water from each breeding site were filtered using 0.22 μm cellulose acetate filter (SIGMA-ALDRICH). Then, the alkalinity and the concentration of sodium (Na^+^), ammonia (NH_4_^+^), nitrates (NO_3_^−^), fluoride (F^−^), magnesium (Mg^2+^), chlorine (Cl^−^), potassium (K^+^), calcium (Ca^2+^), sulphates (SO_4_^2−^), hydrogen carbonate (HCO_3_^−^), and phosphates (PO_4_^3−^) were determined using a standardized ion chromatography spectrometry procedure.

### Molecular Anopheles species identification, insecticide resistance status, and transcriptomic analysis

To determine the presence and proportion of *An. coluzzii* at each breeding site, total genomic DNA was extracted from the first pool of ten larvae stored in 100% ethanol using the DNeasy Extraction Kit (Qiagen, Manchester, UK), according to the manufacturer’s instruction. Species identification of the *An. gambiae* complex was determined based on a ribosomal DNA-based PCR–RFLP assay^60^ The presence of the target site L1014F (kdr-w) mutations was determined using the TaqMan assay, as described in Bass *et* al.^123^.

Breeding sites with higher *An. coluzzii* frequency were selected for gene expression analysis. After collection, the specimens were immediately stored in an RNAlater reagent to preserve RNA. Total RNA was individually isolated from the remaining three pools using the RNeasy Kit (Qiagen) following the manufacturer's instructions. To minimize the variable level of gene expression from one larva to another at the same site, three RNA pools were constructed using the Mini-Elute kit (Qiagen) following the manufacturer's instructions. After purification of the RNA from each pool with the Turbo DNA-free Kit (Qiagen), RNA quality and quantity were assessed using a Nanodrop spectrophotometer (Nanodrop Technologies UK) and Qubit 2.0 Fluorometer (Invitrogen), respectively. RNA was amplified and converted into double-stranded cDNA using the SuperScript III First-Strand Synthesis System (Life Technologies). The PCR protocol developed by Santolomazza^124^ was used to discriminate between *An. coluzzii* and *An. gambiae,* the only two members of the *An. gambiae* complex present at our breeding sites.

Previous studies identified genes with divergent expression profiles in larvae developing in urban breeding sites^50,125^. Ten candidate genes involved in insecticide resistance, detoxification mechanisms and osmoregulation^62,73,119,125^ were selected, and when needed, specific primers were designed using Primer v3 (*bioinfo.ut.ee/primer3-0.4.0/*) (Table S2). Expression levels were estimated following the formula: (Ct _candidate gene_ – Ct _housekeeping gene_)/ Ct _housekeeping gene_, where Ct is the threshold cycle. Moreover, we normalized our analysis using the *RPL19* housekeeping gene^126^. PCR reactions were carried out in 96-well plates (Applied Biosystems). Each well contained 10 µl of Master mix SYBR Green (10X) (Applied Biosystems), 0.2 µl of sense and antisense primers (10 µM), 7.6 µl of distilled H_2_O, and 2 µl of cDNA. Gene expression was quantified using the 7500 Fast Real Time PCR System (Applied Biosystems), and the following amplification conditions: 2 min at 50 °C, 10 min of pre-incubation at 95 °C followed by 40 cycles for 15 s at 95 °C and one minute at 60 °C.

### Statistical analysis

First, a principal components analysis (PCA) was performed with the quantitative variables to ordinate the sites and the urban vs. rural habitats using the package *ade4*^127^. The PCA also allows integrating the significant PCs after selection with the Broken Stick Model as independent variables, thus reducing the risk of multicollinearity and the number of variables^53,61^.

Then, *An. coluzzii* frequency variation (in relation to *An. gambiae*) according to the different physicochemical parameters of the breeding sites and habitats was investigated by building a binomial generalized linear mixed model using the package *lme4*^128^. Response variable was the frequency of *An. coluzzii* (in relation to *An. gambiae*) in larval habitats and explanatory variables were the different physicochemical parameters of the breeding sites. It should be noted that in order to normalize the physicochemical variables, they first underwent a *Boxcox* transformation using the ‘’boxcoxfr’’ function of the *AID* package^129^. The significant interaction between variables was estimated with the Akaike Information Criterion (AIC) for model selection^130^. AIC changes were evaluated when model terms were added or removed using the *dredge* function in the MuMIn package^34^. The model with the lowest AIC was considered the best model. However, the models with a ΔAICC < 2^131^ were retained. Accordingly, multi-model parameter estimates were calculated with the MuMIn package. The ''*Predict*'' function of the *lme4* package and the *plot3D* package were used to determine and describe how the relative *An. coluzzii* frequency (in relation to *An. gambiae*) would evolve according to the habitat type, altitude and PCs. The average expression levels of each gene were compared between rural and urban areas using the Wilcoxon test. In order to investigate the correlation between the general pattern of gene expression and water quality in larval habitats, a coinertia analysis was carried out using the "coinertia" function of the *ade4* package. And the correlogram between genes was done using the "matrix Plot" function of the *GGally* package^132^. All statistical analyses were carried out using the R 3.5.2 software (https://cran.r-project.org).

## Supplementary Information

Supplementary Information 1.

Supplementary Information 2.
