# Supplementary material for: Ecological plasticity to ions concentration determines genetic response and dominance of Anopheles coluzzii larvae in urban coastal habitats of Central Africa
Source: Sci Rep. 2021 Aug 4;11:15781. doi: 10.1038/s41598-021-94258-6 (PMC8338965; doi:10.1038/s41598-021-94258-6)
Supplement: Supplementary file 1 — Supplementary Information 1. [file 41598_2021_94258_MOESM1_ESM.docx]

**SUPPLEMENTARY INFORMATION**

**Ecological plasticity to ions concentration determines genetic response and dominance of *Anopheles coluzzii* larvae in urban coastal habitats of Central Africa.**

***Running title: Coastal urban adaptation of Anopheles coluzzii.***

**Authors**

Neil M. Longo-Pendy^1^, Billy Tene-Fossog^1^, Robert E. Tawedi^2^, Ousman Akone-Ella^1^, Celine Toty^3^, Nil Rahola^3^, Jean-Jacques Braun^2, 4, 5^, Nicolas Berthet^1, 6^, Pierre Kengne^1, 3^, Carlo Costantini^3^ & Diego Ayala^1, 3, &^

**Affiliations**

^1^ CIRMF, Franceville, Gabon.

^2^ Institut de Recherches Géologiques et Minières / Centre de Recherches Hydrologiques, Yaoundé, Cameroon.

^3^ MIVEGEC, Univ Montpellier, CNRS, IRD, Montpellier, France

^4^ Géosciences Environnement Toulouse, Université de Toulouse, CNRS, IRD, Toulouse, France.

^5^ International Joint Laboratory DYCOFAC, IRGM-UY1-IRD, BP 1857, Yaoundé, Cameroon.

^6^ Institut Pasteur, Unité Environnement et Risque Infectieux, Cellule d’Intervention Biologique d’Urgence, Paris, France

**Supplementary figures**


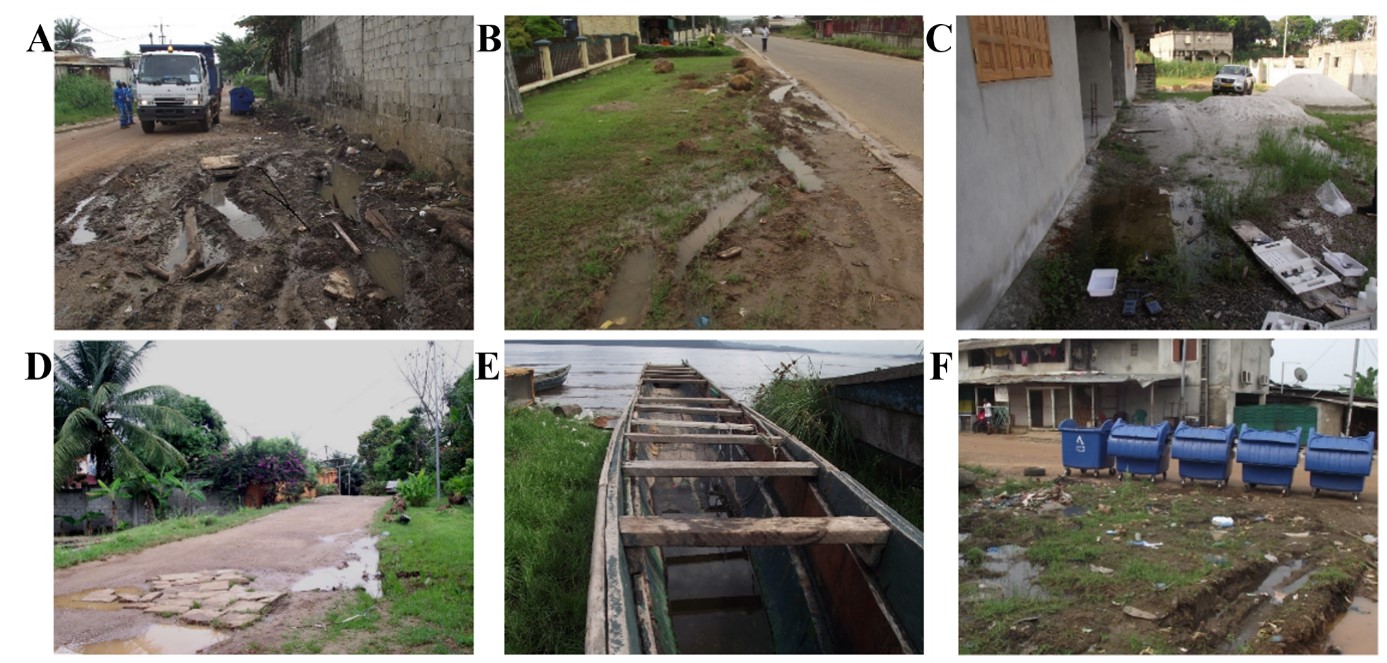


**Figure S1**: Examples of habitats of *An. gambiae* *s.l* larvae in urban and coastal cities of Gabon and Cameroon. (A) Rut in a non-paved secondary road (LBV10). (B) Rut in a platform near the roadway (LBV15). (C) Puddle near a house (LBV1). (D) Puddle formed on a deteriorating roadway (LBV13). (E) Abandoned pirogue on the beach (CCB04). (F) Rut in a rough terrain (LBV18). Photos from Longo-Pendy Neil M.

**Figure S2:** Variation in gene expression among sites. See Table S1

**Figure S3:** Variation in gene expression among localities. See Table S1 and Fig .
